# Hostplant change and paleoclimatic events explain diversification shifts in skipper butterflies (Family: Hesperiidae)

**DOI:** 10.1186/s12862-017-1016-x

**Published:** 2017-08-02

**Authors:** Ranjit Kumar Sahoo, Andrew D. Warren, Steve C. Collins, Ullasa Kodandaramaiah

**Affiliations:** 1 IISER-TVM Centre for Research and Education in Ecology and Evolution (ICREEE), School of Biology, Indian Institute of Science Education and Research, Thiruvananthapuram, Kerala 695 551 India; 2 McGuire Center for Lepidoptera and Biodiversity, Florida Museum of Natural History, University of Florida, PO Box 112710, 3215 Hull Rd., UF Cultural Plaza, Gainesville, FL 32611-2710 USA; 3 African Butterfly Research Institute (ABRI), PO Box 14308 0800, Nairobi, Kenya

**Keywords:** K-Pg, Ecological opportunity, Diversification, Paleocene-Eocene Thermal Maximum, Grass-feeding, Monocot feeding, Insect-hostplant, Coevolution

## Abstract

**Background:**

Skippers (Family: Hesperiidae) are a large group of butterflies with ca. 4000 species under 567 genera. The lack of a time-calibrated higher-level phylogeny of the group has precluded understanding of its evolutionary past. We here use a 10-gene dataset to reconstruct the most comprehensive time-calibrated phylogeny of the group, and explore factors that affected the diversification of these butterflies.

**Results:**

Ancestral state reconstructions show that the early hesperiid lineages utilized dicots as larval hostplants. The ability to feed on monocots evolved once at the K-Pg boundary (ca. 65 million years ago (Mya)), and allowed monocot-feeders to diversify much faster on average than dicot-feeders. The increased diversification rate of the monocot-feeding clade is specifically attributed to rate shifts in two of its descendant lineages. The first rate shift, a four-fold increase compared to background rates, happened ca. 50 Mya, soon after the Paleocene-Eocene thermal maximum, in a lineage of the subfamily Hesperiinae that mostly fed on forest monocots. The second rate shift happened ca. 40 Mya in a grass-feeding lineage of Hesperiinae when open-habitat grasslands appeared in the Neotropics owing to gradual cooling of the atmospheric temperature.

**Conclusions:**

The evolution of monocot feeding strongly influenced diversification of skippers. We hypothesize that although monocot feeding was an intrinsic trait that allowed exploration of novel niches, the lack of extensive availability of monocots comprised an extrinsic limitation for niche exploration. The shifts in diversification rate coincided with paleoclimatic events during which grasses and forest monocots were diversified.

**Electronic supplementary material:**

The online version of this article (doi:10.1186/s12862-017-1016-x) contains supplementary material, which is available to authorized users.

## Background

The remarkable diversity of life is often attributed to either the gradual accumulation of species over long periods of time [1, 2] or to dramatic changes in diversification rates across lineages and time [3, 4]. However, paleontological evidence [3] and phylogenetic comparisons [4] across the tree of life predict the latter scenario as the major cause, where the fluctuations in speciation and extinction rates explain the historical pattern of lineage accumulation. A major cause for these varying rates is rapid diversification promoted by ecological opportunity (EO) [5–8]. EO is generated when a macroevolutionary process affects a lineage in two ways: (i) the lineage evolves the ability to utilize resources otherwise unavailable (by evolutionary innovation; intrinsic factor), and (ii) when new resources become available to the lineage (by colonization or antagonistic extinction or in situ availability of new resource; extrinsic factors) [5–9].

Many comparative studies have shown how rapid diversification is associated with evolutionary innovations ([7–9]; but see [10, 11]). Of particular note is the evolution of herbivory, i.e., shifts from carnivory to herbivory, which has repeatedly elevated diversification rates [12, 13], and this is especially true in the case of insects [13]. Indeed, herbivorous insects comprise about half of all terrestrial eukaryotic species [13], exemplifying the importance of insect-plant interactions in generating the diversity of life [14–16]. Among butterflies, major shifts in hostplant use have led to bursts in diversification rates [17, 18]. For instance, Satyrini butterflies (Subfamily Satyrinae: Tribe Satyrini), which specialise on grasses and include ca. 2200 species, diversified simultaneously with the expansion of grasslands [19]. Therefore, feeding on grasses appears to be closely associated with increased diversification rate in Satyrini.

Paleontological records and comparative phylogenetic analyses indicate that diversification of a taxon is often associated with the extinction of others [20–22]. For instance, nymphalid butterflies (Family Nymphalidae) radiated immediately after the K-Pg extinction (~65 million years ago (Mya)) [23]. Alternatively, in situ availability of new resources can elevate diversification rates of the lineage (see [7, 8]). For instance, paleoclimatic events trigger the appearance and diversification of several groups of animals and plants [24–27], which in turn influence diversification of other taxa. Phytophagous insects, for example, diversified following the evolution of flowering plants ([22, 28]; but see [29, 30]). Furthermore, colonization of a novel geographic area, newly formed islands for instance, are often associated with increased diversification [31–33].

Thus, evidence supporting the role of EO in accelerating diversification largely comes from analyses of distinct macroevolutionary processes that represent either intrinsic or extrinsic factors. However, theory predicts that both availability of resources due to extrinsic factors and the intrinsic ability to utilize them are necessary to generate EO [7–9]. For instance, a lineage capable of utilizing a new but scarce resource may undergo rapid diversification when those resources become abundant in situ. Although intrinsic and extrinsic factors of EO are often presented independently, their role in generating EO are not mutually exclusive; and, as the aforementioned predictions suggest, the factors promoting EO may appear one after another over a phylogeny, but EO is generated only when all the factors become available to the lineage. However, this possibility is not explored in detail for any group of organisms.

We here investigate the pattern of appearance of intrinsic and extrinsic factors of EO and their effect on diversification in a highly speciose but hitherto ignored group of butterflies - the skippers (Family Hesperiidae). The skippers comprise ca. 4000 species distributed among ca. 567 genera [34]. About 50% of skippers feed on monocots during larval stages. We compiled a 10-gene dataset of 7726 bp (base pair) from 290 genera representing all the known major clades. Based on estimated times of divergence, we checked whether skippers have experienced rapid shifts in diversification rates, indicative of adaptive radiation(s). To test the hypothesis that the rate shifts have occurred in response to ecological opportunity, we specifically investigate whether (i) rate shifts are associated with feeding preferences of the lineages, (ii) the K-Pg extinction had an impact on diversification rate, (iii) the diversification pattern reflects the biogeographic distribution of lineages, and (iv) paleoclimate has influenced diversification rate.

## Methods

### Phylogenetic and molecular dating analyses

We used a concatenated dataset of nine nuclear genes and one mitochondrial gene for the divergence time estimation. Our dataset was built upon the previous work [34], to which we added 34 specimens, resulting in a dataset of 290 genera that accounts for nearly 60% of known skipper genera. Many skipper genera have highly restricted distributions and comprise very few species, making them logistically very challenging to sample. Therefore, despite the massive field effort of several collaborators worldwide spanning more than a decade, we were unable to achieve a more comprehensive sampling of the taxa. However, we believe we have included representatives of all the major clades.

A previous study [34] with 270 skipper samples showed that there are two equally plausible topologies of hesperiid relationships. Hence, we first investigated whether the existence of the contrasting topologies (see [34]) has any effect on the age estimates of its major clades. We used 105 branch-optimized trees obtained from independent Maximum Likelihood (ML) analysis of the previously published dataset [34] with gene partitions. We estimated ultrametric trees using the PATHd8 method [35], which uses the mean path length algorithm with correction for a molecular clock, implemented in the program PATHd8 v1.0 [35]; and assigned an arbitrary time unit of one to the crown age of the ingroup to estimate relative times of divergence for nodes. We then calculated the distribution of relative age estimates of the deeper clades from these ultrametric trees.

We used the software BEAST v2.4.2 [36] on the CIPRES Science Gateway [37] to simultaneously estimate phylogenetic relationships and times of divergences for our current dataset. Sequences of two species from Hedylidae, which is the sister family to Hesperiidae [38], were acquired from Genbank and treated as outgroups. We estimated the most appropriate partitioning scheme for the data matrix using TIGER v1.02 (Tree Independent Generation of Evolutionary Rates) [39] and the nucleotide substitution models for the partitions by PartitionFinder v1.1.1 [40]. We assumed a relaxed clock model that allowed branch lengths to vary as per an uncorrelated lognormal distribution, and assigned a Birth-Death Process as the tree prior. We assigned an exponential distribution of mean 10.0 as the prior for the hyperparameter ‘ucldMean’. Priors of all other parameters were kept at their default values.

We used the previously estimated age ranges (81–114 Mya) [38] to calibrate the split between Hesperiidae and Hedylidae (uniform distribution), along with a recently described fossil [41] to constraint the minimum stem age of Hesperiinae (25 Mya; log-normal distribution). Posteriors of the parameters were estimated from two independent runs of 50 million generations each. We checked the convergence of independent runs from the distribution of their log-likelihood scores using the program Tracer v1.6 [42]. To confirm proper mixing of samples from every generation, we checked the Effective Sample Size of all parameters after discarding the initial 20% of trees as burnin. The tree files, after discarding burnin, were combined using LogCombiner v2.4.5 and the parameter values were annotated to the Maximum Clade Credibility (MCC) tree using TreeAnnotater v2.4.5 (a part of BEAST v2.4.5 package; [36]).

### Diversification analysis

For subsequent analyses, we retained only one randomly chosen representative species for each genus if multiple species for that genus were available. We estimated gamma (γ) statistic [43] from the MCC tree using the R (v3.2.3; [44]) package Laser v2.4.1 [45]. The gamma statistic indicates whether internal nodes are closer to the root (γ < 0) or to the tips (γ > 0) of the tree than expected under a constant rate model (γ = 0). We accounted for incomplete taxon sampling by adjusting the critical value for gamma using the Monte Carlo Constant Rates (MCCR) test [43].

We generated a Lineage Through Time (LTT) plot from the MCC tree. To evaluate the pattern of lineage accumulation in the empirical LTT plot, we generated 1000 trees under the birth-death model with 290 taxa using the R package TreeSim v2.2 [46]. These trees were then used to construct a mean LTT curve with 95% confidence interval and compared with the empirical LTT curve. We evaluated the fit of the lineage accumulation on the MCC tree to different models of diversification [47] using hierarchical likelihood ratio test and Akaike Information Criteria (AIC) in the R package Laser v2.4.1 [45].

We calculated the diversification rates of higher level clades following the method-of-moments estimator for stem-group ages [48] and checked using Phylogenetic Generalized Least Squares (PGLS) [49] in the R package APE v3.5 [50] whether clade age or diversification rate predicts species richness. PGLS accounts for phylogenetic non-independence of clades while modeling regression between the parameters.

We employed the program BAMM v2.5.0 [51] to model the diversification rate shifts. This is a Bayesian approach that describes the number and locations of rate shifts as posterior distributions. We specified the sampling probability as the proportion of representative species in our dataset out of total known species in each tribe or subfamily. To check for the prior sensitivity of BAMM to the number of rate shifts detected, we performed multiple BAMM analyses with varying priors (1, 5 and 10) for expected rate shifts. We ran the analyses for 5 million generations each and sampled every 5000 generations. Since priors had little effect on the results, we used one as the rate shift prior during the final analysis, performed with four independent chains of 20 million generations each and sampled every 20,000 generations. After removing the first 20% of generations as burnin, the number of rate shifts and rate shift configurations were estimated using the R package BAMMtools v2.1.0 [52]. We note that although BAMM is very popular, there has been recent debate about the reliability of the method. In particular, the likelihood function and the prior used in BAMM have been argued to be flawed [53], but [54] later argued that these problems do not apply to most datasets. Rather than relying solely on BAMM, we have adopted multiple modelling approaches and we believe our results are robust to the potential flaws of BAMM (see Discussion for more detail).

In another approach, we estimated trait-specific diversification rates considering hostplants as binary characters - monocots or dicots. About 16% of the taxa in our dataset feed on magnoliids or both monocot and dicot; we assigned them as data unavailable. The character states for each tip in the phylogeny were compiled from multiple sources (Additional file 1: Appendix S1). We applied the Binary State Speciation and Extinction model (BiSSE) [55] in the R package diversitree v0.9.7 [56]. BiSSE calculates speciation, extinction and transition rates for each assigned character state. We accounted for the incomplete sampling in our dataset by providing the total proportion of missing taxa in our dataset. We used a uniform prior probability and 10,000 MCMC steps to estimate the posterior probability distribution of each parameter.

Nevertheless, the presence of unmeasured factors could potentially influence the diversification pattern estimated for the states of the observed trait over a phylogeny, and hence can lead to erroneous inference when using SSE (State dependent Speciation and Extinction) models [57, 58]. Thus, we also tested whether shifts in diversification rates correlate with the shift in feeding habit or any unmeasuerd factors, by applying Hidden State Speciation and Extinction model (HiSSE) [58].

### Character mapping

We mapped the known biogeographic distribution of the sampled taxa onto the phylogeny using the online tool Interactive Tree Of Life (iTOL) v3 [59]. For each missing genus, we randomly assigned the distribution to a sampled genus from the same tribe or subfamily. The geographic range of genera was compiled from various sources (Additional file 1: Appendix S1) and was divided into six biogeographic regions – Neotropical, Nearctic, Palearctic, Afrotropical, Oriental and Australian.

We also annotated the phylogeny with the larval hostplant data of sampled genera using iTOL v3 [59] and accounted for the hostplants of missing genera in the manner as was done for geographic ranges above. Based on information about larval hostplant use, we divided the genera into three broad categories – dicots, monocots and magnoliids. Additionally, the monocots were subdivided into Poales, Arecales, Zingiberales and Asparagales.

## Results

Although a previous study [34] indicated two equally likely tree topologies for higher relationships in skipper butterflies, after scaling those topologies in relative times with the crown age of skippers assigned to one unit time, we observed that the relative times of divergences across the contrasting topologies were similar for all major clades, except Euschemoninae, Eudaminae and two clades of Pyrginae (Additional file 1: Figure S1). To assess the influence of topological uncertainties on diversification analyses, we performed all diversification analyses additionally on 100 randomly selected posterior trees from the dating analysis, which showed no such influence.

### Diversification rates and pattern

**Fig. 1:**
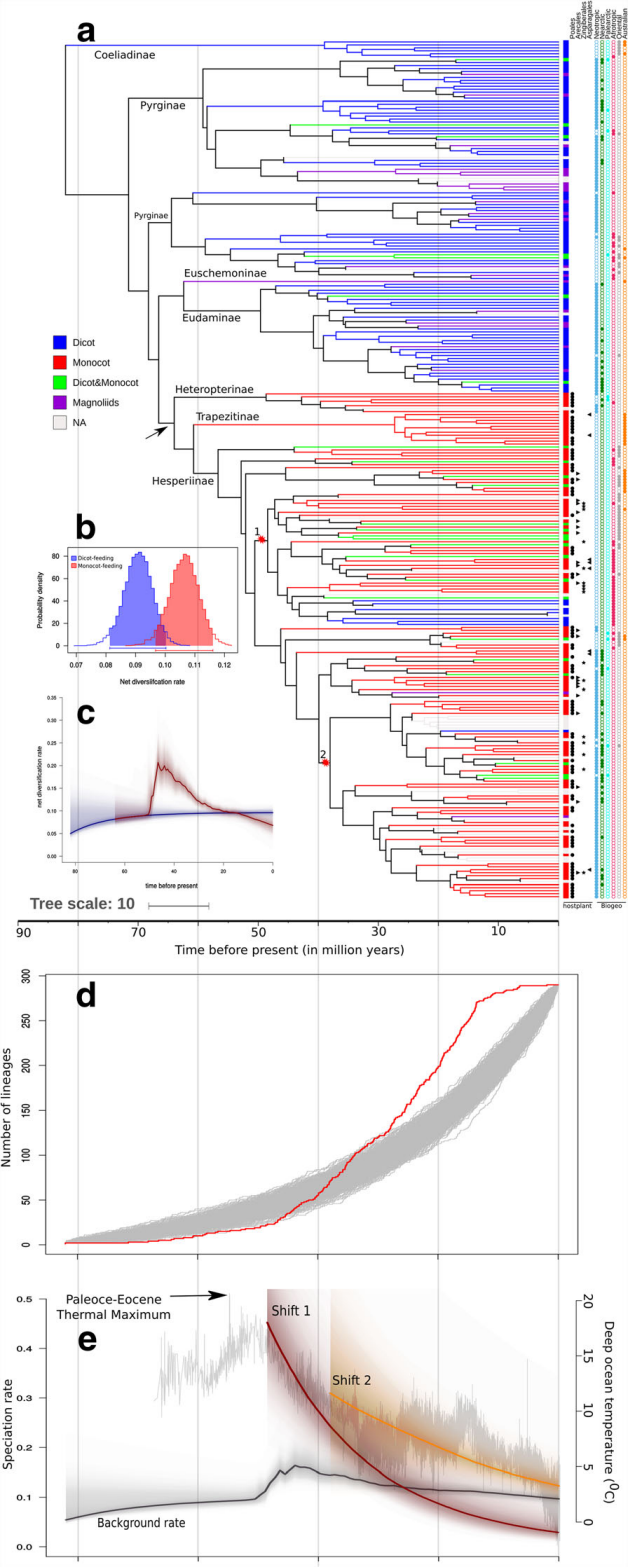
Diversification rates across the hesperiid phylogeny and the comparison of rates with paleoclimatic events. A more detailed figure with names of genera is shown in Additional file 2. **a** The hesperiid time tree mapped with hostplant data and geographic distributions. The names at nodes represent subfamilies. The terminal branches are colored based on the broad category of hostplant use. Monocot feeding taxa are additionally shown with the known categories of plants (*black Circle*: Poales; *Outward triangle*: Arecales; *Star*: Zingiberales; *Inward triangle*: Asparagales) they feed upon. *Coloured circles* represent known distributions of the taxa (a *filled circle* indicates presence in the area). The *arrow* indicates the shift from dicot to monocot feeding. The *red stars* at nodes indicate the points of diversification rate shifts (numbered as shift 1 and 2) from the BAMM analysis. **b** Posterior distributions from the BiSSE analysis for net diversification rates of monocot- and dicot-feeding hesperiids. Lines below each distribution are 95% confidence intervals. **c** Net diversification rate from the BAMM analysis for the dicot- (*blue*) and monocot-(*red*) feeding lineages. **d** The LTT curve (in *red*) of the MCC tree superimposed on the LTT curves (*in grey*) from 1000 trees simulated under constant diversification rate for 290 taxa. **e** The change in paleoclimatic temperature (as in [93]) (in *grey*) plotted using the R package RPADNA v1.2 [94]. This climatic plot is superimposed with the mean speciation rate (along with the posterior distributions) for the whole hesperiid phylogeny (in *dark grey*) and the speciation rates of the lineages those experienced rate shifts (shift 1 and 2 as in (**a**))

The MCC tree indicates that Hesperiidae began diversifying in the late Cretaceous ca. 82 Mya, when Coeliadinae diverged from the rest of the family. Based on the Monte Carlo Constant Rates (MCCR) test, internal nodes were significantly closer to the root than expected, indicative of a decrease in net diversification rate over time (gamma = −13.88, critical gamma = −12.72 at *p* = 0.004). The LTT plot deviated significantly from a simulated curve generated under constant diversification rate with incomplete taxon sampling (Fig. 1d), indicating heterogeneity in diversification rate through time. Moreover, a model of diversification specifying change in rate best fits the lineage accumulation on the MCC tree (Additional file 1: Table S1).

The pattern of variation in diversification rates was also reflected in the BAMM analysis accounting for extant species richness. There was a slow and continuous increase in the net diversification rate up to 50 Mya (Fig. 1e; black curve), after which the slope of the curve increased steadily indicating an increase in the rate. After ~40 Mya, the diversification rate gradually declined.

This disparity in diversification rates across the phylogeny explains the species richness of higher-level clades (PGLS: *r* = 0.87; *p* < 0.0001). The trait-independent analysis in BAMM estimated two major shifts in diversification rate over the phylogeny (Fig. 1a) (basal rate: 0.10, rate at 1 st shift = 0.48 and 2nd shift = 0.24), both within the subfamily Hesperiinae. We note that while the probability for two rate shifts on the phylogeny is 0.83, the point estimates for those shifts have varying probabilities, i.e. 0.9 for 1st shift and 0.6 for 2nd shift, as evident from credibility set of rate shifts (Additional file 1: Figure S2).

### Influence of the K-Pg event

The LTT plot shows that the diversification rate slowed down around K-Pg boundary (~ 65 Mya), suggesting either slower speciation rate or lineage extinctions (Fig. 1d). The rate of lineage accumulation remained low up to ca. 50 Mya, after which the rate suddenly increased.

### Hostplant use and Biogeography

Mapping of the larval hostplant data onto the phylogeny (Fig. 1a) indicated that the early hesperiid lineages fed on dicots. Around ca. 65 Mya, one lineage shifted to monocot feeding and later this lineage gave rise to three subfamilies including the most speciose subfamily Hesperiinae.

The posterior distribution from the BAMM analysis indicates that the net diversification rate of the monocot-feeding clade was higher than that of the dicot-feeding clades (Fig. 1c). The differential diversification rates across the monocot- and dicot-feeding clades were corroborated in the BiSSE analysis (Fig. 1b), where the higher diversification rate of the monocot-feeding lineages corresponded to a higher speciation rate although extinction rates did not differ between dicot- and monocot-feeding groups (Additional file 1: Figure S3). Topological uncertainties had no major influence on these estimations, as the AIC value from the MCC tree analysis is close to the average of the AIC values from 100 randomly selected posterior trees under a full model analysis (Additional file 1: Figure S4). However, a comparison of the SSE models (Additional file 1: Table S2) indicated that the model with hidden (unmeasured) states of the trait best fits the diversification pattern.

We did not find any strong associations between diversification rate shifts and biogeographic patterns (Fig. 1a). However, the mapping illustrated that the first rate shift happened in a lineage distributed across the Oriental and Afrotropical regions. The second rate shift occurred in a Neotropical lineage.

## Discussion

We present the most comprehensive phylogenetic hypothesis of hesperiid butterflies, including ca. 300 taxa. Based on this phylogeny, we infer the macroevolutionary history of the group in relation to historical events and coevolutionary interactions with their hostplants.

Skippers started evolving ca. 90 Mya, when dicots were the dominant plants [60], and early lineages of the family diversified on these plants. Evolution of monocot feeding ca. 65 Mya provided the intrinsic ability to utilize otherwise unavailable ecological resources. This novel interaction with the environment resulted a higher net diversification rate of the monocot-feeding group (Fig. 1b, c). The robustness of this correlation (monocot feeding ~ diversification) to fundamentally different approaches of analyses (BAMM, and BiSSE; see Fig. 1b, c) lends further support to the higher diversification of monocot-feeding group. Although the monocot-feeding lineage represented <20% of the lineages at the K-Pg boundary (~65 Mya), it includes ca. 50% of extant species.

However, model comparisons indicated a possible influence of unknown factors on the estimated diversification pattern suggesting that the association between monocot-feeding and diversification rate must be influenced by certain unmeasured factors. Interestingly, we found that the change to monocot feeding did not increase diversification immediately – the rate shift, a ca. four-fold increase compared to the background rate, occurred ca. 50 Mya (Fig. 1a, e) within the subfamily Hesperiinae. Although the intrinsic ability to utilize monocots provided new capability for niche exploration, the low availability of monocots probably imposed an extrinsic limitation that did not allow high rates of diversification initially. The earliest monocot-feeding lineages specialized on grasses (Fig. 1a); the limited availability of open grassland habitats may have also imposed a limitation on diversification. The rate shift occurred soon after the Paleocene–Eocene Thermal Maximum (PETM) ca. 55–56 Mya, a period when the earth’s temperature increased dramatically as a result of addition of carbon to the oceans and the atmosphere [61, 62].

The PETM followed less-severe warming periods up to early Eocene (~ 50 Mya) mainly due to shuffling of carbon between atmosphere and ocean [63]. These warm periods are associated with diversification of forest dwelling monocots and also experienced compositional changes in insect fauna, as evidenced by fossil records [64, 65]. Eocene fossils indicate elevated levels of insect herbivory (leaf damage frequency) and greater insect herbivore diversity per hostplant (leaf damage type) [28, 64]. Thus, the rapid radiation of skippers ca. 50 Mya coincides with the historical diversification pattern of herbivorous insects in the Eocene, although the selective forces behind these radiations are not known [65].

The increase in diversification beginning ca. 50 Mya happened in lineages distributed in the Oriental and Afrotropical regions. This was followed by a downward shift in diversification rate, during the late Eocene to Oligocene, possibly due to saturation of ecological niches in these biogeographic areas. The diversification rate again increased (about two-fold compared to the background rate) ca. 40 Mya in a Neotropical grass-feeding (Poales) lineage (Fig. 1a, e). This shift coincides with the appearance of open-habitat grasses in the Neotropics when the atmospheric temperature gradually decreased [66–68]. Therefore, the emergence of open-habitat grasslands appears to provide the extrinsic factor of EO for the diversification of skipper butterflies. Although grasses became dominant in Neotropics during the Miocene (~ 20 Mya) [68], this increased availability of grasses, interestingly, had little impact on hesperiids.

Our study suggests that the extrinsic availability of resources can modulate the potential of intrinsic ability in generating EO for rapid diversification. The diversification pattern in Satyrinae butterflies [19] is consistent with this hypothesis. In Satyrinae, the ability to feed on grasses appeared early in the lineage; however, rapid diversification occurred in one lineage – the tribe Satyrini – when grasses became abundant during Oligocene (~ 33–26 Mya) [19].

Although grass feeding has been thought to be an important evolutionary innovation that is closely tied to rapid radiations of most herbivorous insects [69], the mechanistic basis of increased speciation rates due to grass feeding is unclear. Diffuse coevolution [70–72] between insects and their hostplants, which has been widely shown to increase insect speciation rates [13, 73, 74], has been attributed to the pressure on herbivorous insects to specialize. As first postulated by Ehrlich and Raven [14], plants evolve chemical defenses against herbivores [75], which forces herbivores to specialize [76], leading to a coevolutionary arms race [15, 16]. Episodes of generalization and specialization can then lead to increased speciation rates [77, 78]. However, grass-feeding insects tend to be broad generalists, with rare examples of narrow specialists (see [79, 80]). This is likely because most grasses are expected to rely predominantly on physical defenses such as silicifaction [81–83] (also see [84, 85]). Although chemical defenses in the form of secondary metabolites [86, 87] and fungal-mediated toxins [88, 89] have been reported in grasses (also see [90, 91]), the extent to which these chemicals confer defense against herbivory are still obscure and limited to few taxa.

Yet, satyrines and skippers, the major butterfly groups that feed on grasses, both appear to have radiated rapidly as a result of grass feeding. We opine that this is likely not because of the classical insect-hostplant coevolutionary arms race postulated by Ehlrich and Raven [14]. Rather, the opportunity to invade grasslands (savannahs) could have been the key. The dominance of grasslands over forest communities may have increased rates of allopatric speciation.

We did not find evidence for the effect of the K-Pg boundary on the diversification of skipper butterflies, unlike in nymphalid butterflies that radiated explosively immediately after this event [23]. However, the evolution of monocot feeding coincided with the K-Pg event. We hypothesize that the catastrophic events during this period led to the extinction of competing herbivores on monocots, and thus allowed the inclusion of monocots in the hostplant repertoire of skippers.

### Potential methodological concerns

#### BiSSE

Although hugely popular, BiSSE is known to perform poorly when the number of tips is low, and when the tip-ratio bias (ratio of numbers of tips with different character states) is high [92]. Davis et al. [92] concluded that BiSSE results should be inferred with extreme caution when the phylogeny included fewer than 300 terminals and/or when fewer than 10% of species are of one character state. Although tip-ratio bias is unlikely to be a problem in our dataset, we acknowledge that the number of tips is a concern. However, the main result from BiSSE, i.e., increased diversification of monocot feeders compared to that of dicot feeders, is also corroborated by BAMM, a fundamentally different modelling approach. Therefore we believe that the result is robust.

#### BAMM

Moore et al. [53] argued that BAMM is strongly affected by the priors specified, and that the estimates of diversification rate parameters are unreliable (but see [54]). To check the effect of priors on the BAMM results, we performed multiple analyses with varying priors for rate shifts, but found that priors had no effect on the number or positions of rate shifts detected.

Therefore, we have confidence in the main results presented here.

## Conclusions

We have shown that the ability of skipper butterflies to feed on monocots evolved at the K-Pg boundary and provided a novel intrinsic EO that eventually allowed monocot-feeders to have a higher diversification rate compared to dicot-feeders. The diversification rate (a four-fold increase compared to background rates) itself shifted ca. 50 Mya, soon after the PETM, in a lineage of the subfamily Hesperiinae. We attribute this delayed increase in diversification to the limited availability of monocots, which comprised an extrinsic limitation for niche exploration. There was another increase in diversification rate (a ca. two-fold increase) in a Neotropical Hesperiinae lineage ca. 40 Mya, coinciding with the appearance of grassland communities in the region. We suggest that grass feeding allowed rapid radiations in the two major groups of grass-feeding butterflies - skippers and satyrines - not through the classical insect-hostplant coevolutionary arms race, but by enhancing the chances of allopatric speciation.

## Additional files

Additional file 1: “Hostplant change and paleoclimatic events explain diversification shifts in skipper butterflies (Family: Hesperiidae).” **Figure S1.** Comparison of ultrametric trees of contrasting topologies calibrated using one time unit as the crown age. **Table S1.** Fitting of models of diversification to the lineage accumulation pattern in MCC tree. **Figure S2.** 95% credibility shift configurations with the configuration frequencies summarized with the posteriors from the BAMM analysis. **Figure S3.** Posterior distributions of speciation and extinction rates of dicot- and monocot-feeding lineages from the BiSSE analysis. **Figure S4.** Graphs showing the relative positions of AIC values from the BiSSE analysis of the MCC tree on the distribution of the AIC values from 100 random posterior trees from the dating analysis. **Table S2.** Exploring the effect of hidden states on the diversification rate estimation. **Appendix S1.** Data References. (PDF 510 kb)

Additional file 2: The hesperiid time tree. (PDF 4563 kb)
