# Supplementary material for: Hostplant change and paleoclimatic events explain diversification shifts in skipper butterflies (Family: Hesperiidae)
Source: BMC Evol Biol. 2017 Aug 2;17:174. doi: 10.1186/s12862-017-1016-x (PMC5541431; doi:10.1186/s12862-017-1016-x)
Supplement: Supplementary file 1 — “Hostplant change and paleoclimatic events explain diversification shifts in skipper butterflies (Family: Hesperiidae).” Figure S1. Comparison of ultrametric trees of contrasting topologies calibrated using one time unit as the crown age. Table S1. Fitting of models of diversification to the lineage accumulation pattern in MCC tree. Figure S2. 95% credibility shift configurations with the configuration frequencies summarized with the posteriors from the BAMM analysis. Figure S3. Posterior distributions of speciation and extinction rates of dicot- and monocot-feeding lineages from the BiSSE analysis. Figure S4. Graphs showing the relative positions of AIC values from the BiSSE analysis of the MCC tree on the distribution of the AIC values from 100 random posterior trees from the dating analysis. Table S2. Exploring the effect of hidden states on the diversification rate estimation. Appendix S1. Data References. (PDF 510 kb) [file 12862_2017_1016_MOESM1_ESM.pdf]

Additional file 1 for "Hostplant change and paleoclimatic events explain diversification shifts in skipper butterflies (Family: Hesperiidae)."
(Detailed legends are given along with respective figure or table)

Figure S1: Comparison of ultrametric trees of contrasting topologies calibrated using one time unit as the crown age.

Table S1: Fitting of models of diversification to the lineage accumulation pattern in MCC tree.

Figure S2: 95% credibility shift configurations with the configuration frequencies summarized with the posteriors from the BAMM analysis.

Figure S3: Posterior distributions of speciation and extinction rates of dicot- and monocot-feeding lineages from the BiSSE analysis.

Figure S4: Graphs showing the relative positions of AIC values from the BiSSE analysis of the MCC tree on the distribution of the AIC values from 100 random posterior trees from the dating analysis.

Table S2: Exploring the effect of hidden states on the diversification rate estimation.

Appendix S1: Data References

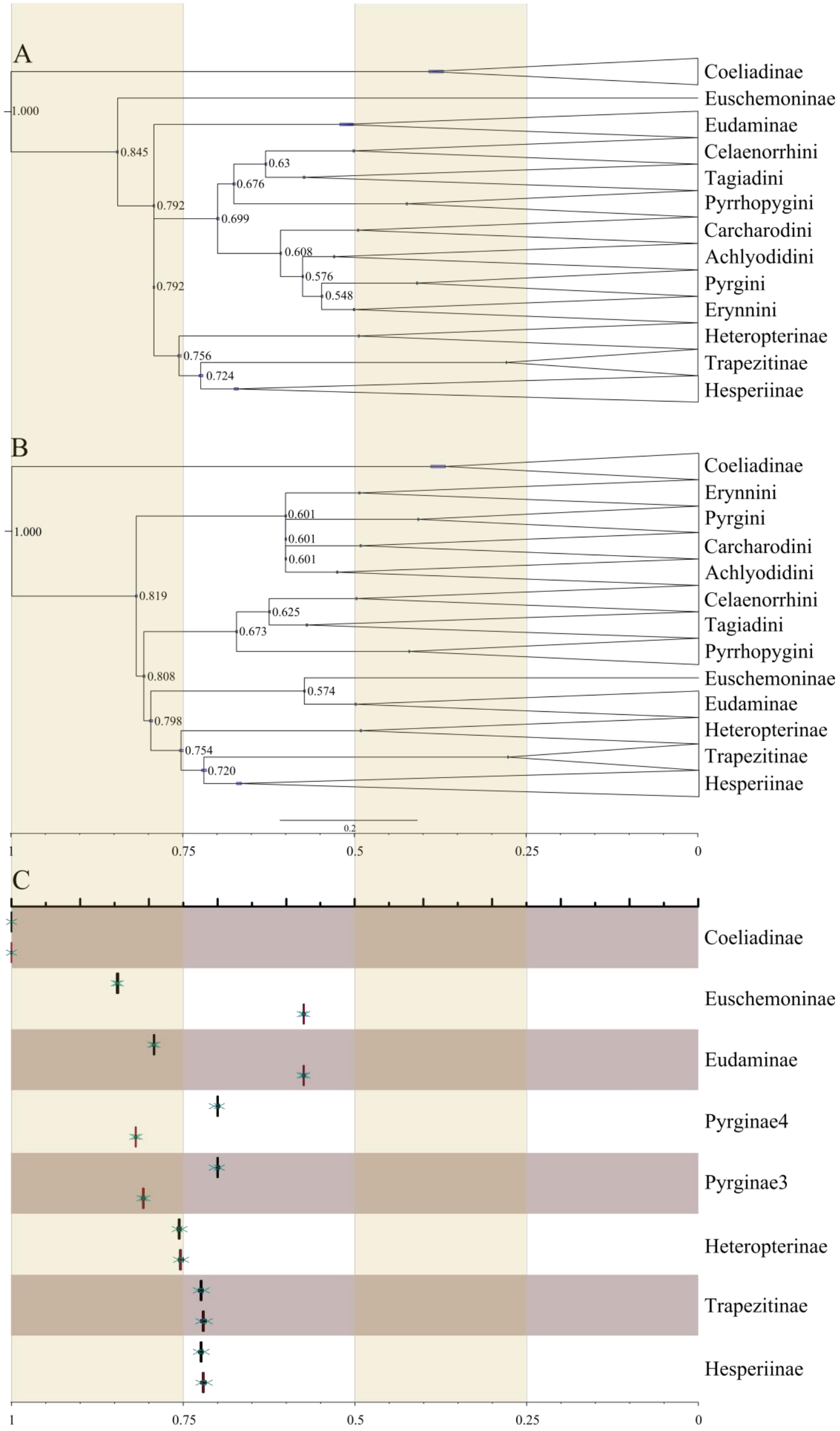

Figure S1: Comparison of ultrametric trees of contrasting topologies (A) and (B) calibrated using one time unit as the crown age of the skipper phylogeny. These two trees are random samples from the ultrametric trees derived from the calibration of 105 ML trees from the concatenated dataset from Sahoo et al. (2016) with gene-partitions. Node values represent the median age calculated from 48 and 57 time trees respectively for (A) and (B). The distribution of all node ages estimated from the contrasting topologies (black and red for A and B respectively; cross mark represents the average value) are in (C). N.B: Pyrginae4 is the clade containing four tribes of Pyrginae and Pyrginae3 is the clade containing 3 tribes.

Table S1: Comparison of different models of diversification fitted to the diversification pattern in the MCC tree. Pure Birth is a pure birth or Yule model of diversification, Birth Death is a constant birth death model of diversification, SPVAR considers a variable speciation rate and constant extinction rate, EXVAR considers a variable extinction rate and constant speciation rate, yule2rate assumes an abrupt change in diversification rate. DDX and DDL fit exponential and logistic variants of the density-dependent speciation rate model respectively. AIC = Akaike information criteria; logLik = log-likelihood; λ, μ are speciation and extinction rates.

	Pure Birth	Birth Death	SPVAR	EXVAR	yule2rate	DDX	DDL
logLik	92.994	92.994	165.266	91.441	202	139.474	136.947
AIC	-183.987	-185.987	-324.531	-176.882	-398.01	-274.948	-269.894
λ	0.034	0.034	0.43	0.035	0.044	0.497	0.054
μ	-	0	0.001	0.001	0.001	-	-

Best Constant Rate Model = pureBirth; AIC = -183.987
Best Rate Variable Model = yule2rate; AIC = -398.01
The test statistic delta AIC = 214.023

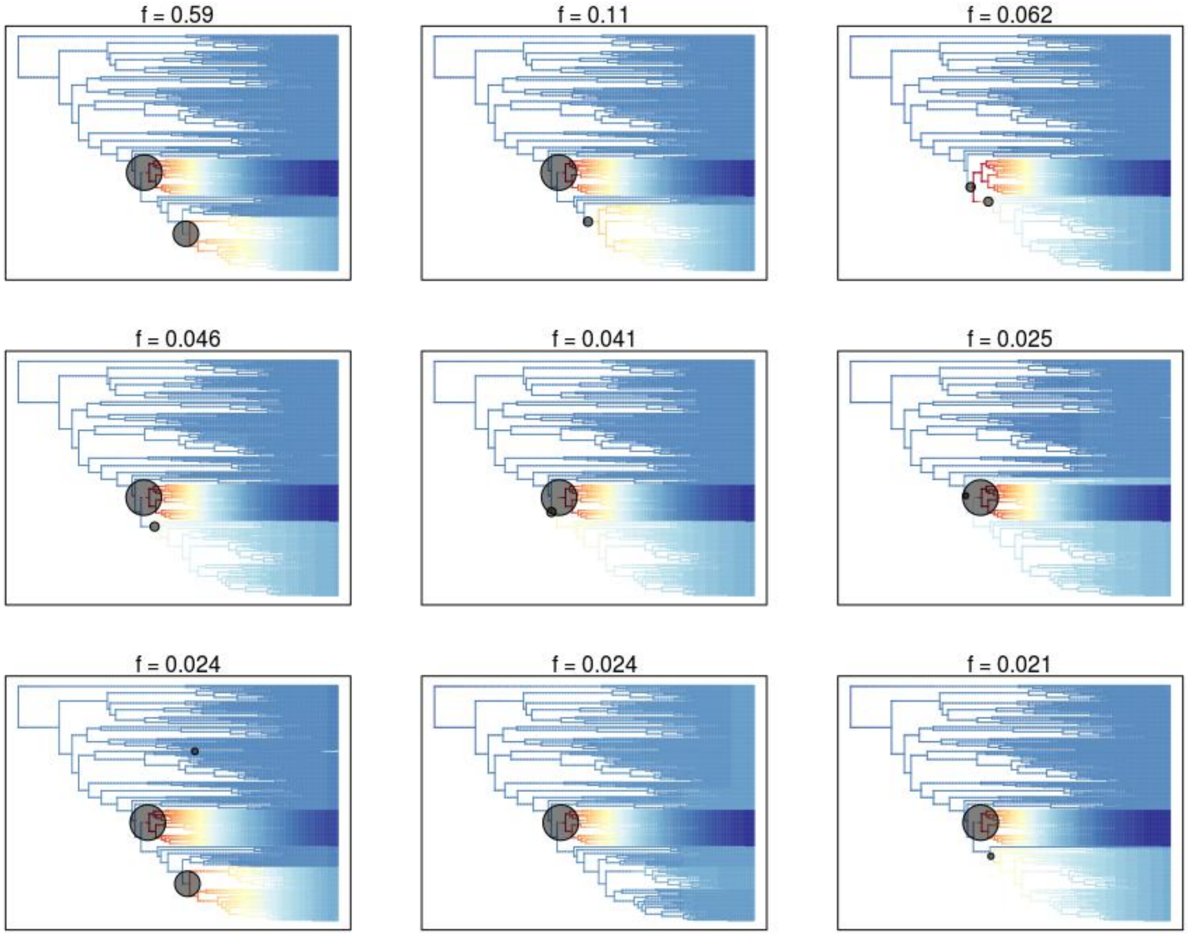

Figure S2: 95% credibility shift configurations with the configuration frequencies (mentioned on the top of each tree) summarized from the BAMM analysis. Size of the circles at the nodes correspond to the marginal probability of the shifts at the positions.

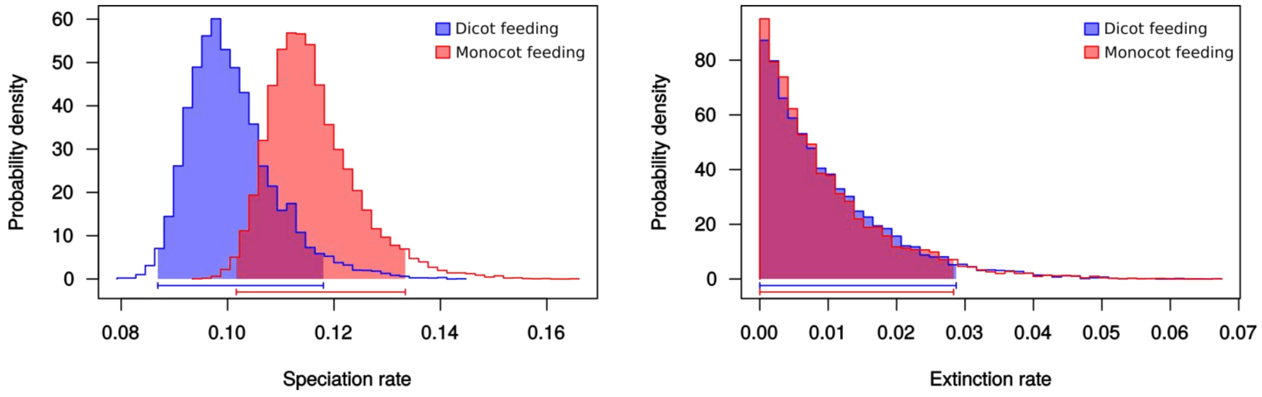

Figure S3: The posterior distribution of the speciation and extinction rates of dicot- and monocot- feeding lineages from BiSSE analysis. Lines below each distribution are 95% confidence intervals.

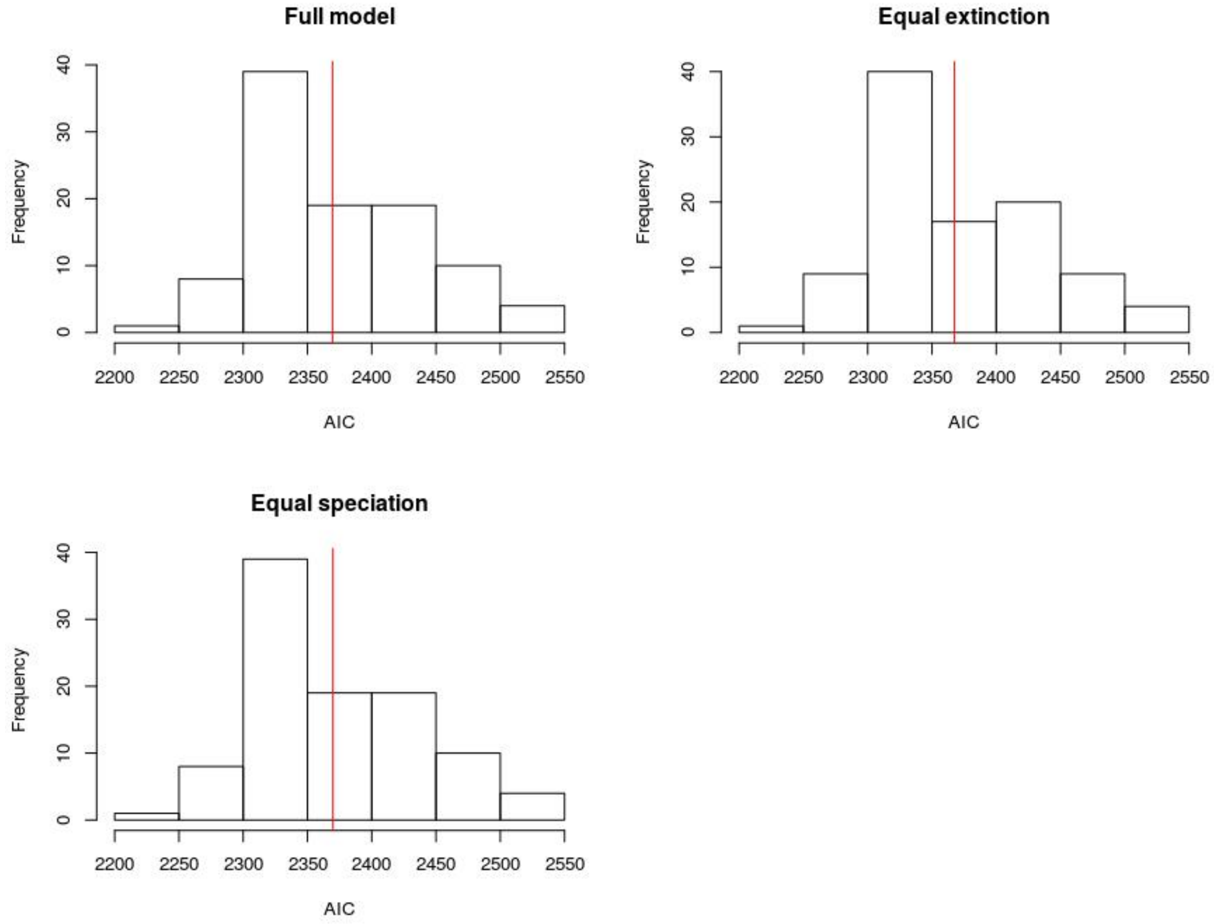

Figure S4: Graphs showing the relative positions of AIC (Akaike information criteria) values from the BiSSE analysis of the time tree on the distribution of the AIC values from 100 posterior trees from the dating analysis. All analyses were performed for equal sampling frequency (290/3941) for both character states.

Table S2: Exploring the effect of hidden state on the diversification rate estimation. Null model on the HiSSE framework allows trait independent change in diversification rate. The rate changes as a function of an unobserved hidden trait. Null model on the BiSSE framework allows equal rates across the states and no hidden character. In trait dependent BiSSE model, either the diversification rate changes with the trait or one of the trait may associate with a hidden state. Comparison of these shows that the model where an unobserved hidden state is associated with monocot feeding lineages gets the lowest AIC value. AIC = Akaike information criteria; logLik = log-likelihood.

	logLik	AIC	ΔAIC
null HiSSE	-859.8965	1729.793	0
full HiSSE	-848.3926	1736.785	6.992
null BiSSE	-944.5391	1897.078	167.285
BiSSE (trait dependent)	-943.8176	1899.635	169.842
BiSSE (hidden state associated with monocot feeding)	-869.1325	1758.265	28.472

Appendix S1: Data References

1. Warren A.D., Ogawa J.R., Brower A.V. (2009) Revised classification of the family Hesperiidae (Lepidoptera: Hesperioidea) based on combined molecular and morphological data. *Systematic Entomology* 34(3):467-523. **(This paper has a compiled list of larval hostplant information along with their references)**
2. Robinson G.S., Ackery P.R., Kitching I.J., Beccaloni G.W., Hernández L.M. (2010) HOSTS—a Database of the World's Lepidopteran Hostplants. Natural History Museum, London. Available at http://www.nhm.ac.uk/hosts. Last accessed 20 October 2016.
3. Janzen, D. H. and Hallwachs, W. (2016) Caterpillar rearing voucher database for the Area de Conservacion (ACG) in northwestern Costa Rica. Available at: http://janzen.sas.upenn.edu/. Last accessed 04 December 2016.
4. Greeney H.F., Warren A.D. (2003) Notes on the life history of Eantis thraso (Hesperiidae: Pyrginae) in Ecuador. *Journal of the Lepidopterists' society,* 57(1),43-46.
5. Cock M.J.W. (2015) Observations on the Biology of Skipper Butterflies in Trinidad, West Indies: Urbanus, Astraptes and Narcosius (Hesperiidae: Eudaminae). *Living World, Journal of The Trinidad and Tobago Field Naturalists' Club,* 01-14
6. Cock M.J.W. (2014) Observations on the Biology of Skipper Butterflies in Trinidad, Trinidad and Tobago: Phocides, Chioides, Typhedanus, and Polythrix (Hesperiidae: Eudaminae). *Living World, Journal of The Trinidad and Tobago Field Naturalists' Club,* 1-11.
7. Cock M.J.W., Alston-Smith S. (2013) Udranomia spp. (Lepidoptera: Hesperiidae: Eudaminae) in Trinidad, West Indies. *Living World, Journal of The Trinidad and Tobago Field Naturalists' Club,* 29-31.
8. Cock M.J.W. (1981) The Skipper butterflies (Hesperiidae) of Trinidad. Part 1: Introduction and Pyrrhopyginae. *Living World, Journal of The Trinidad and Tobago Field Naturalists' Club,* 52-56.
9. Cock M.J.W. (1984) The Skipper butterflies (Hesperiidae) of Trinidad. Part 3: Pyrginae (first section). *Living World, Journal of The Trinidad and Tobago Field Naturalists' Club,* 38-42.
10. Cock M.J.W. (1986) The Skipper butterflies (Hesperiidae) of Trinidad. Part 4: Pyrginae (second section). *Living World, Journal of The Trinidad and Tobago Field Naturalists' Club,* 33-47.
11. Cock M.J.W. (1988) The Skipper butterflies (Hesperiidae) of Trinidad. Part 5: Pyrginae genera group C concluded. *Living World, Journal of The Trinidad and Tobago Field Naturalists' Club,* 24-31.
12. Cock M.J.W., Alston-Smith S (1990) The Skipper butterflies (Hesperiidae) of Trinidad. Part 6: Pyrginae genera group D. *Living World, Journal of The Trinidad and Tobago Field Naturalists' Club,* 25-35.
13. Cock M.J.W. (1991) The Skipper butterflies (Hesperiidae) of Trinidad. Part 7: Genera group E. *Living World, Journal of The Trinidad and Tobago Field Naturalists' Club*: 46-56.
14. Cock M.J.W. (1996) The Skipper butterflies (Hesperiidae) of Trinidad. Part 8: Genera group E (second section). *Living World, Journal of The Trinidad and Tobago Field Naturalists' Club,* 27-37.
15. Cock M.J.W. (1998) The Skipper butterflies (Hesperiidae) of Trinidad. Part 9: Genera group E concluded (third section) with a description of a new species of *Clito*. *Living World, Journal of The Trinidad and Tobago Field Naturalists' Club,* 33-45.

16. Cock M.J.W. (2000) The Skipper butterflies (Hesperiidae) of Trinidad. Part 10: Pyrginae completed; genera groups F and G. *Living World, Journal of The Trinidad and Tobago Field Naturalists' Club,* 49-71.
17. Cock M.J.W. (2003) The Skipper butterflies (Hesperiidae) of Trinidad. Part 11, Hesperiinae, genera group O. *Living World, Journal of The Trinidad and Tobago Field Naturalists' Club,* 14-48.
18. Cock M.J.W. (2004) The Skipper butterflies (Hesperiidae) of Trinidad. Part 12, Heteroptinae, genera group H and Hesperiinae genera group N. *Living World, Journal of The Trinidad and Tobago Field Naturalists' Club,* 23-25.
19. Cock M.J.W. (2005) The Skipper butterflies (Hesperiidae) of Trinidad. Part 13, Hesperiinae genera group K. *Living World, Journal of The Trinidad and Tobago Field Naturalists' Club,* 23-47.
20. Cock M.J.W. (2006) The Skipper butterflies (Hesperiidae) of Trinidad. Part 14, Hesperiinae genera group L. *Living World, Journal of The Trinidad and Tobago Field Naturalists' Club,* 8-26.
21. Cock M.J.W. (2007) The Skipper butterflies (Hesperiidae) of Trinidad. Part 15, Hesperiinae genera group M. *Living World, Journal of The Trinidad and Tobago Field Naturalists' Club,* 38-56.
22. Cock M.J.W. (2009) The Skipper Butterflies (Hesperiidae) of Trinidad Part 16, Hesperiinae, Genera Group J, Vettius - Naevolus. *Living World, Journal of The Trinidad and Tobago Field Naturalists' Club,* 11-31.
23. Cock M.J.W. (2010) The Skipper Butterflies (Hesperiidae) of Trinidad Part 17, Hesperiinae, Anthoptini and the Remainder of Evans' Genera Group I. *Living World, Journal of The Trinidad and Tobago Field Naturalists' Club,* 11-30.
24. Cock M.J.W. (2011) The Skipper Butterflies (Hesperiidae) of Trinidad Part 18, Hesperiinae, Moncini: eight genera of relatively distinctive species, Callimormus, Eutocus, Artines, Flaccilla, Phanes, Monca, Vehilius and Parphorus. *Living World, Journal of The Trinidad and Tobago Field Naturalists' Club,* 14-36
25. Cock M.J.W. (2013) The Skipper Butterflies (Hesperiidae) of Trinidad. Part 19. Hesperiinae, Moncini: the Remaining Genera with Pale Spots: Cymaenes, Cobalopsis, Arita, Lerema, Morys and Tigasis. *Living World, Journal of The Trinidad and Tobago Field Naturalists' Club,* 20-40
26. Cock M.J.W. (2013) The Skipper Butterflies (Hesperiidae) of Trinidad. Part 20. Hesperiinae, Moncini: the Remaining Genera of Mostly Unmarked Brown Species: Eutocus, Eprius, Mnasicles, Methionopsis, Sodalia, Thargella, Nastra, Mnasilus, Mnasitheus and Papias. *Living World, Journal of The Trinidad and Tobago Field Naturalists' Club,* 1-18
27. Cock M.J.W. (2010) Observations on the biology of Afro–tropical Hesperiidae (Lepidoptera) principally from Kenya. Part 1. Introduction and Coeliadinae. *Zootaxa,* 2547, 1-63.
28. Cock M.J.W., Congdon T.C.E. (2011) Observations on the biology of Afro–tropical Hesperiidae (Lepidoptera) principally from Kenya. Part 2. Pyrginae: Tagiadini. *Zootaxa,* 2893, 1-66.
29. Cock M.J.W., Congdon T.C.E. (2011) Observations on the biology of Afro–tropical Hesperiidae (Lepidoptera) principally from Kenya. Part 3. Pyrginae: Celaenorrhinini. *Zootaxa,* 3033, 1-67.
30. Cock M.J.W., Congdon T.C.E. (2012) Observations on the biology of Afro–tropical Hesperiidae (Lepidoptera) principally from Kenya. Part 4. Hesperiinae: Aeromachini and Baorini. *Zootaxa,* 3438, 1-42.

31. Cock M.J.W., Congdon T.C.E. (2013) Observations on the biology of Afro–tropical Hesperiidae (Lepidoptera). Part 5. Hesperiinae *incertae sedis*: dicotyledon feeders. *Zootaxa,* 3724, 1-85.
32. Cock M.J.W., Congdon T.C.E., Collins S.C. (2014) Observations on the biology of Afro–tropical Hesperiidae (Lepidoptera). Part 6. Hesperiinae *incertae sedis*: palm feeders. *Zootaxa,* 3831, 1-61.
33. Cock M.J.W., Congdon T.C.E. (2014) Observations on the biology of Afro–tropical Hesperiidae (Lepidoptera). Part 7. Hesperiinae *incertae sedis*: grass and bamboo feeders. *Zootaxa,* 3872(4), 301-354.
34. Cock M.J.W., Congdon T.C.E., Collins S.C. (2015) Observations on the biology of Afrotropical Hesperiidae (Lepidoptera). Part 8. Hesperiinae *incertae sedis*: Dracaena feeders. *Zootaxa,* 3985(3), 301-348.
35. Cock M.J.W., Congdon T.C.E., Collins S.C. (2016) Observations on the biology of Afrotropical Hesperiidae (Lepidoptera). Part 9. Hesperiinae *incertae sedis*: Zingiberales feeders, genera of unknown biology and an overview of the Hesperiinae *incertae sedis*. *Zootaxa,* 4066(3), 201-247.
36. Cock M.J.W. (2016) Observations on the biology of Afrotropical Hesperiidae (Lepidoptera) with particular reference to Kenya. Part 10. Pyrginae, Carcharodini. *Zootaxa*, 4173(4), 301-350.
37. Hesperiidae data base from http://www.nic.funet.fi. Available at: http://www.nic.funet.fi/pub/sci/bio/life/insecta/lepidoptera/ditrysia/hesperioidea/hesperiidae/ Last accessed 08 October 2016.
