# Supplementary material for: Hostplant change and paleoclimatic events explain diversification shifts in skipper butterflies (Family: Hesperiidae)
Source: BMC Evol Biol. 2017 Aug 2;17:174. doi: 10.1186/s12862-017-1016-x (PMC5541431; doi:10.1186/s12862-017-1016-x)
Supplement: Supplementary file 2 — The hesperiid time tree. (PDF 4563 kb) [file 12862_2017_1016_MOESM2_ESM.pdf]

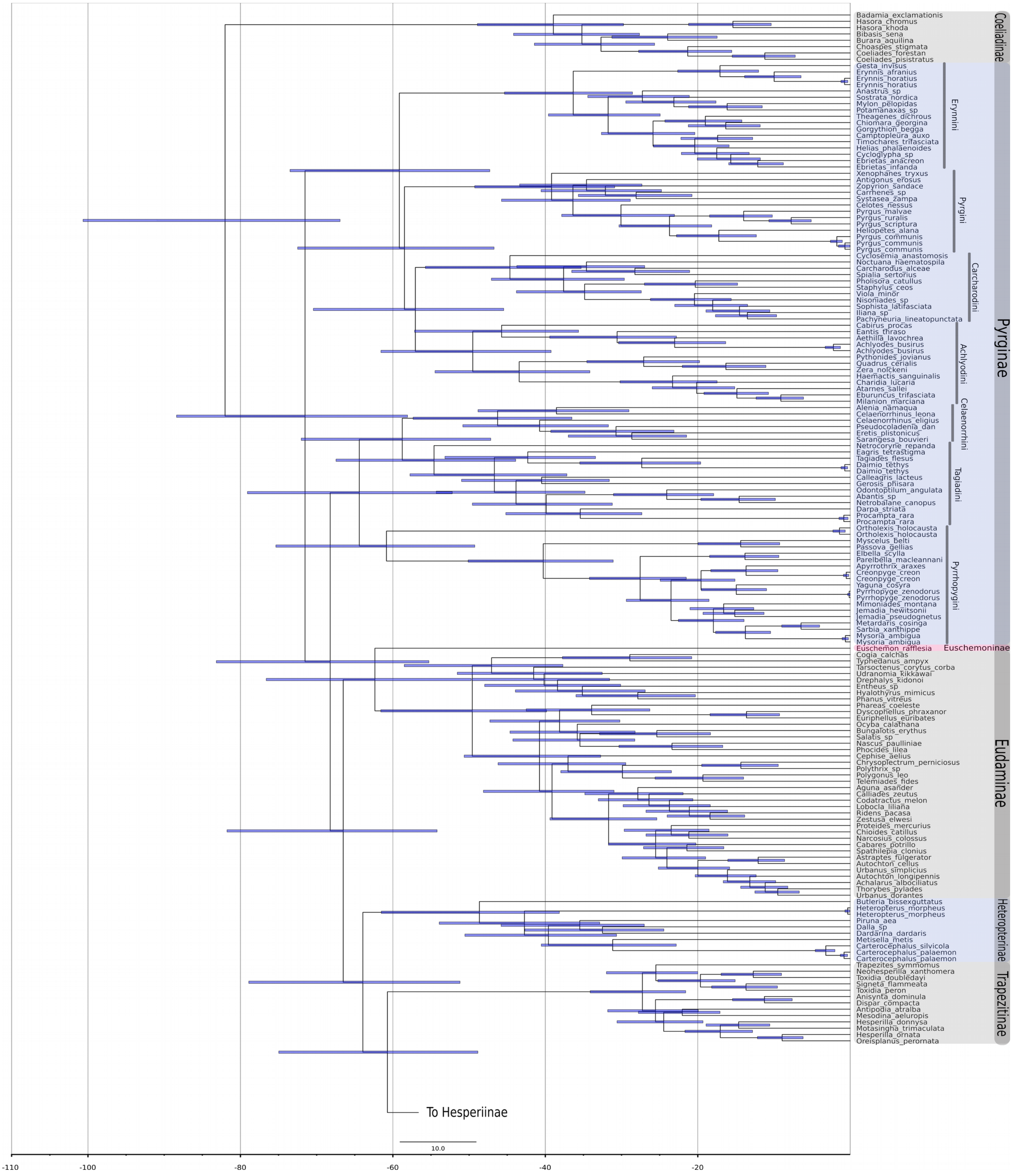

Badamia_exclamationis
Hasora_chromus
Hasora_khoda
Bibasis_sena
Burara_aquilina
Choaspes_stigmata
Coeliades_forestan
Coeliades_pisistratus
Gesta_invisus
Erynnis_afranius
Erynnis_horatius
Erynnis_horatius
Anastrus_sp
Sostrata_nordica
Mylon_pelopidas
Potamanaxas_sp
Theagenes_dichrous
Chiomara_georgina
Gorgythion_begga
Camptopleura_auxo
Timochares_trifasciata
Helias_phalaenoides
Cycloglypha_sp
Ebrietas_anacreon
Ebrietas_infanda
Xenophanes_tryxus
Antigonus_erosus
Zopyrion_sandace
Carrhenes_sp
Systasea_zampa
Celotes_nessus
Pyrgus_malvae
Pyrgus_ruralis
Pyrgus_scriptura
Heliopetes_alana
Pyrgus_communis
Pyrgus_communis
Pyrgus_commnis
Cyclosemia_anastomosis
Noctuana_haematospila
Carcharodus_alceae
Spialia_sertorius
Pholisora_catullus
Staphylus_ceos
Viola_minor
Nisoniades_sp
Sophista_latifasciata
Iliana_sp
Pachyneuria_lineatopunctata
Cabirus_procas
Eantis_thraso
Aethilla_lavochrea
Achlyodes_busirus
Achlyodes_busirus
Pythonides_jovianus
Quadrus_cerialis
Zera_nolckeni
Haemactis_sanguinalis
Charidia_lucaria
Atarnes_sallei
Eburuncus_trifasciata
Milanion_marciana
Alenia_namaqua
Celaenorrhinus_leona
Celaenorrhinus_eligius
Pseudocoladenia_dan
Eretis_plistonicus
Sarangesa_bouvieri
Netrocoryne_repanda
Eagris_tetrastigma
Tagiades_flesus
Daimio_tethys
Daimio_tethys
Calleagris_lacteus
Gerosis_phisara
Odontoptilum_angulata
Abantis_sp
Netrobalane_canopus
Darpa_striata
Procampta_rara
Procampta_rara
Ortholexis_holocausta
Ortholexis_holocausta
Myscelus_belti
Passova_gellias
Elbella_scylla
Parelbella_macleannani
Apyrrothrix_araxes
Creonpyge_creon
Creonpyge_creon
Yaguna_cosyra
Pyrrhopyge_zenodorus
Pyrrhopyge_zenodorus
Mimoniades_montana
Jemadia_hewitsonii
Jemadia_pseudognetus
Metardaris_cosinga
Sarbia_xanthippe
Mysoria_ambigua
Mysoria_ambigua
Euschemon_rafflesia
Cogia_calchas
Typhedanus_ampyx
Tarsoctenus_corytus_corba
Udranomia_kikkawai
Drephalys_kidonoi
Entheus_sp
Hyalothyrus_mimicus
Phanus_vitreus
Phareas_coeleste
Dyscophellus_phraxanor
Euriphellus_euribates
Ocyba_calathana
Bungalotis_erythus
Salatis_sp
Nascus_paulliniae
Phocides_lilea
Cephise_aelius
Chrysoplectrum_perniciosus
Polythrix_sp
Polygonus_leo
Telemiades_fides
Aguna_asander
Calliades_zeutus
Codatractus_melon
Lobocla_liliana
Ridens_pacasa
Zestusa_elwesi
Proteides_mercurius
Chioides_catillus
Narcosius_colossus
Cabares_potrillo
Spathilepia_clonius
Astraptes_fulgerator
Autochton_cellus
Urbanus_simplicius
Autochton_longipennis
Achalarus_albociliatus
Thorybes_pylades
Urbanus_dorantes
Butleria_bissexguttatus
Heteropterus_morpheus
Heteropterus_morpheus
Piruna_aea
Dalla_sp
Dardarina_dardaris
Metisella_metis
Carterocephalus_silvicola
Carterocephalus_palaemon
Carterocephalus_palaemon
Trapezites_symmomus
Neohesperilla_xanthomera
Toxidia_doubledayi
Signeta_flammeata
Toxidia_peron
Anisynta_dominula
Dispar_compacta
Antipodia_atralba
Mesodina_aeluropis
Hesperilla_donnysa
Motasingha_trimaculata
Hesperilla_ornata
Oreisplanus_peronata
Coeliadinae
Erynnini
Pyrgini
Carcharodini
Achlyodini
Celaenorrhini
Tagiadini
Pyrrhopygini
Pyrginae
Euschemoninae
Eudaminae
Heteropterinae
Trapezitinae
To Hesperiinae
10.0
-110
-100
-80
-60
-40
-20

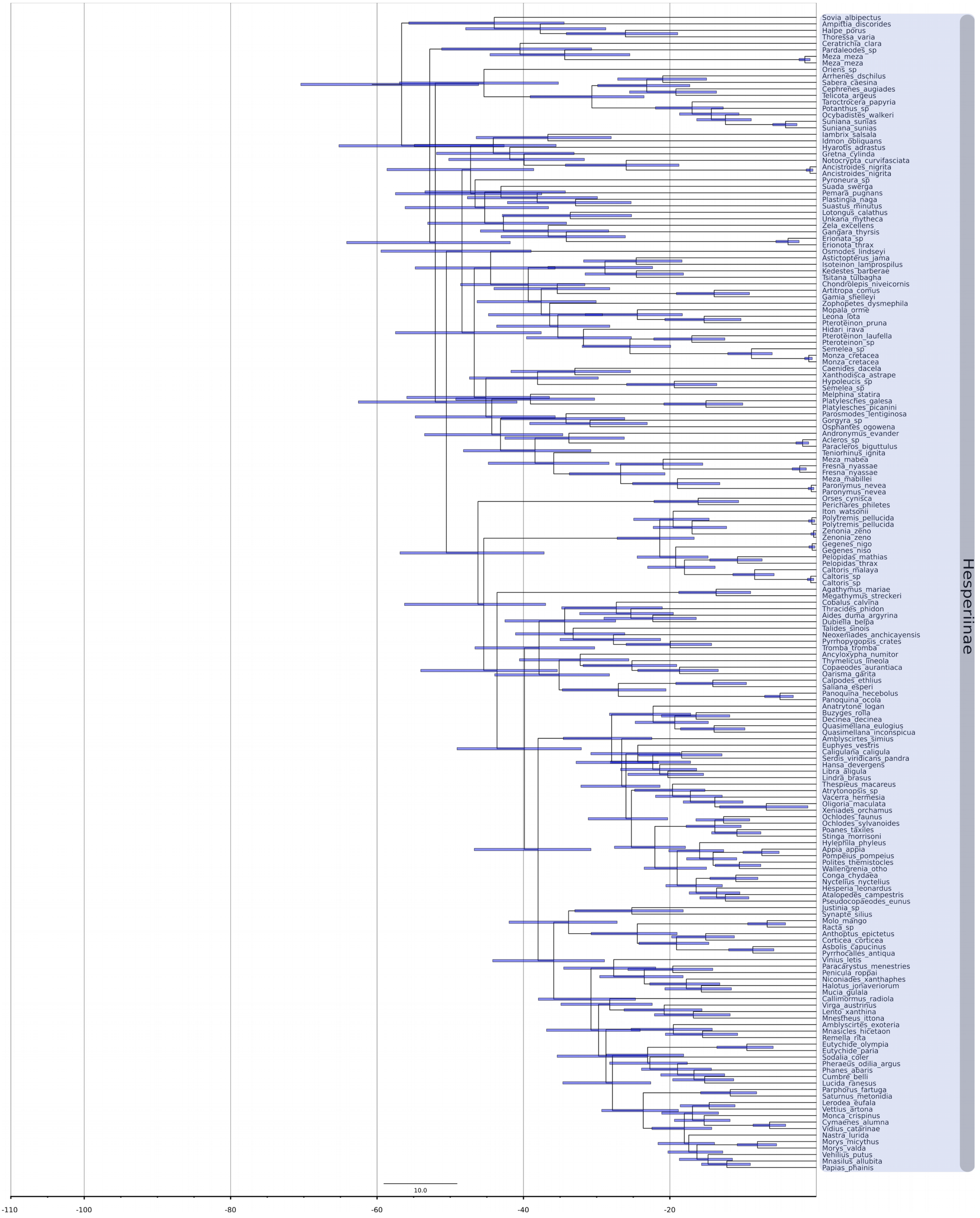
Sovia_albipectus
Ampittia_discorides
Halpe_porus
Thoressa_varia
Ceratrichia_clara
Pardaleodes_sp
Meza_meza
Meza_meza
Oriens_sp
Arrhenes_dschilus
Sabera_caesina
Cephrenes_augiades
Telicota_argeus
Taractrocera_papyria
Potanthus_sp
Ocybadistes_walkeri
Suniana_sunias
Suniana_sunias
Iambrix_salsala
Idmon_obliquans
Hyarotis_adrastus
Gretna_cylinda
Notocrypta_curvifasciata
Ancistroides_nigrita
Ancistroides_nigrita
Pyroneura_sp
Suada_swerga
Pemara_pugnans
Plastingia_naga
Suastus_minutus
Lotongus_calathus
Unkana_mytheca
Zela_excellens
Gangara_thyrsis
Erionota_sp
Erionota_thrax
Osmodes_lindseyi
Astictopterus_jama
Isoteinon_lamprospilus
Kedestes_barberae
Tsitana_tulbagha
Chondrolepis_niveicornis
Artitropa_comus
Gamia_shelleyi
Zophopetes_dysmephila
Mopala_orma
Leona_lota
Pteroteinon_pruna
Hidari_irava
Pteroteinon_laufella
Pteroteinon_sp
Semelea_sp
Monza_cretacea
Monza_cretacea
Caenides_dacela
Xanthodisca_astrape
Hypoleucis_sp
Semelea_sp
Melphina_statira
Platylesches_galesa
Platylesches_picanini
Parosmodes_lentiginosa
Gorgyra_sp
Osphantes_ogowena
Andronymus_evander
Acleros_sp
Paracleros_biguttulus
Teniorhinus_ignita
Meza_mabea
Fresna_nyassae
Fresna_nyassae
Meza_mabillei
Paronymus_nevea
Paronymus_nevea
Orses_cynisca
Perichares_philetes
Iton_watsonii
Polytremis_pellucida
Polytremis_pellucida
Zenonia_zeno
Zenonia_zeno
Gegenes_nigo
Gegenes_niso
Pelopidas_mathias
Pelopidas_thrax
Caltoris_malaya
Caltoris_sp
Caltoris_sp
Agathymus_mariae
Megathymus_streckeri
Cobalus_calvina
Thracides_phidon
Aides_duma_argyrina
Dubiella_belpa
Talides_sinois
Neoxeniades_anchicayensis
Pyrrhopygopsis_crates
Tromba_tromba
Ancyloxypha_numitor
Thymelicus_lineola
Copaeodes_aurantiaca
Oarisma_garita
Calpodes_ethlius
Saliana_esperi
Panoquina_hecebolus
Panoquina_ocola
Anatrytone_logan
Buzyges_rolla
Decinea_decinea
Quasimellana_eulogius
Quasimellana_inconspicua
Amblyscirtes_simius
Euphyes_vestris
Caligulana_caligula
Serdis_viridicans_pandra
Hansa_devergens
Libra_aligula
Lindra_brasus
Thespieus_macareus
Atrytonopsis_sp
Vacerra_hermesia
Oligoria_maculata
Xeniades_orchamus
Ochlodes_faunus
Ochlodes_sylvanoides
Poanes_taxiles
Stinga_morrisoni
Hylephila_phyleus
Appia_appia
Pompeius_pompeius
Polites_themistocles
Wallengrenia_otho
Conga_chydaea
Nyctelius_nyctelius
Hesperia_leonardus
Atalopedes_campestris
Pseudocopaeodes_eunus
Justinia_sp
Synapte_silius
Molo_mango
Racta_sp
Anthoptus_epictetus
Corticea_corticea
Asbolis_capucinus
Pyrrhocalles_antiqua
Vinius_letis
Paracarystus_menestries
Penicula_roppai
Niconiades_xanthaphes
Halotus_jonaveriorum
Mucia_gulala
Callimormus_radiola
Virga_austrinus
Lento_xanthina
Mnestheus_ittona
Amblyscirtes_exoteria
Mnasicles_hicetaon
Remella_rita
Eutychide_olympia
Eutychide_paria
Sodalia_coler
Pheraeus_odilia_argus
Phanes_abaris
Cumbre_belli
Lucida_ranesus
Parphorus_fartuga
Saturnus_metonidia
Lerodea_eufala
Vettius_artona
Monca_crispinus
Cymaenes_alumna
Vidius_catarinae
Nastra_lurida
Morys_micythus
Morys_valda
Vehilius_putus
Mnasilus_allubita
Papias_phainis
Hesperiinae
10.0
-110
-100
-80
-60
-40
-20
